# Spatial assessment of drought severity in Cape Town area, South Africa

**DOI:** 10.1016/j.heliyon.2019.e02148

**Published:** 2019-07-31

**Authors:** I.R. Orimoloye, O.O. Ololade, S.P. Mazinyo, A.M. Kalumba, O.Y. Ekundayo, E.T. Busayo, A.A. Akinsanola, W. Nel

**Affiliations:** a Centre for Environmental Management, University of the Free State, Bloemfontein, South Africa; b Department of Geography and Environmental Science, University of Fort Hare, Alice, South Africa; c Department of Meteorology and Climate Science, Federal University of Technology Akure, Nigeria; d School of Energy and Environment, City University of Hong Kong, Hong Kong Special Administrative Region

**Keywords:** Natural hazards, Ecology, Environmental impact assessment, Remote sensing, Environmental impact, Severity, Remote sensing, Assessment, Drought, Vegetation health

## Abstract

In recent decades, drought has been identified as part of the several regular climate-related hazards happening in many African countries including South Africa, often with devastating implications on food security. Studies have shown that the earth temperature has increased over the recent years which can trigger drought occurrences and other climate-related hazards. Drought occurrence is principally a climate-related event that cannot be totally effaced though it can be managed. This study is aimed at appraising drought severity in Cape Town area, South Africa using Geographic Information System (GIS) and remotely sensed data obtained from United States Geological Survey (USGS) database between the years 2014 and 2018. The study revealed that the land use dynamics witnessed drastic changes where vegetation, water body and bare surface decreased from 2095 to 141 km^2^, 616 to 167 km^2^ and 2337 to 1381km^2^ respectively while built up and sparse vegetation increased from 5301 to 8191 km^2^ and 7382–7854 km^2^ during the period. Vegetation health and drought severity of the study area was assessed using vegetation indices and Normalized Drought Dryness Index (NDDI). The result reveals that Normalized Difference Water Index (NDWI) and other vegetation indices decreased considerably more in recent years (2017 and 2018) which might have triggered drought events during the period compared to the other years (2014–2016). Furthermore, the spatial trend of land surface temperature (LST) and NDDI increased in recent years with NDDI values ranging between moderate drought and severe drought threshold. Consequently, if the increment persists, it can lead to adverse impacts such as food insecurity, land degradation and environmental health deterioration. Evidently, this study reveals the current state of vegetation health regarding drought severity in the area using remotely sensed data.

## 1 Introduction

Remote multispectral and hyperspectral measurements have been an imperative source of data for drought and vegetation dynamics assessment. Several multispectral vegetation indices (VIs) have been employed to appraise growing vegetation attributes in recent decades (Adam et al., 2010; Viña et al., 2011; Yang et al., 2012). These indices are essential in terms of analytical orders of reflectance in the blue, green, near-infrared and red spectral bands which have been recognized to be connected with green vegetation factors. These factors include water index, leaf area index and drought index (Rhee et al., 2010; Viña et al., 2011), canopy cover (Garbulsky et al., 2011), the fraction of absorbed and reflected land surface temperature and surface radiation (Orimoloye et al., 2018b). Restrictions, however, have existed due to the effect of external factors for example, soil and dead material setting, solar and viewing geometry as well as meteorological event, all of which pose a challenge in carrying out a proper assessment.

The impacts of drought have been assessed through the estimation of green vegetation in drought-affected areas either as long or short-term dryness using vegetation indices such as Normalized Difference Water Index (NDWI), Soil Adjusted Total Vegetation Index (SATVI), Normalized Difference Vegetation Index (NDVI), Land Surface Temperature (LST) and Normalized Drought Dryness Index (NDDI) in the prior research, particularly in an arid or semi-arid and mild Mediterranean ecosystem where vegetation is sporadic (Gu et al., 2007; Orimoloye et al., 2018c). Sparse or scanty vegetation in grasslands alludes to the dead part of the grasslands such as fallen litter and standing dry grasses aggregated from previous years either by natural or anthropogenic practices (Leslie and Richman, 2018; Otto et al., 2018).

Drought has been identified by several researchers to be the most difficult yet less likely to be understood of all natural disasters in-term of mitigation and influencing human activities when compared to some other climate-related events (Mann and Gleick, 2015; Lemos et al., 2016; Orimoloye et al., 2018a). Thus, it is a weighty and cumbersome environmental factor in the world's climatic zones including South Africa (Mann and Gleick, 2015; Lemos et al., 2016). It has played an imperative role in many sectors such as health and agriculture. Drought always starts with water scarcity for domestic and agricultural use, thus affecting streams, soil moisture, groundwater, ecosystems, water bodies, wetlands and human action (Orimoloye et al., 2018c). This may lead to the recognition of various forms of droughts (agricultural, meteorological, ecological and socioeconomic), which connotes the perspectives of various components on water dearth. Moreover, among these components, agriculture can be more influenced adversely by the onset of droughts event as a result of its dependence on soil moisture and water resources conservation during several stages of crop growth.

The technique of multispectral analysis of vegetation health has been employed to characterize drought severity (Alshaikh, 2015; Dutta et al., 2015; Trisasongko et al., 2015; Khosravi et al., 2017; Zhang et al., 2017a,b) with an adoptable outcome. The advent of RS has imparted immensely to the use of digital and global data with computers and software applications such as ArcGIS, ENVI, ILWIS and QGIS for analyzing, processing, managing and monitoring this remotely sensed information to aid drought assessment and other hazards as well as providing solutions to the challenges facing local and global sustainable advancement (Piao et al., 2003). RS and GIS are features of earth observation science and have contributed an advanced system for arranging, analyzing, manipulating and storing the information about the spatial components including drought and vegetation health. Hence, remotely sensed data and GIS techniques have been utilized in recent decade to monitor urban features as well as the environmental changes (Adefisan et al., 2015; Onamuti et al., 2017; Stephen et al., 2017; Orimoloye et al., 2018b, 2018c).

The dynamics of soil water under drought situations could result to changes in soil spectral reflectance identified on RS information (Gao et al., 2011). Studies have suggested that an increase in soil humidity in open surfaces such as bare or cultivated lands lead to a decrease in soil reflectivity (Garbulsky et al., 2011; Zargar et al., 2011). Several indices have been utilized by different studies to monitor, quantify and map droughts subject to the environmental and climatological factors as well as the indices extracted from the satellite images. Furthermore, several indices such as Vegetation Condition Index, VCI (Quiring and Ganesh, 2010), Normalized Difference Vegetation Index, NDVI (Swain et al., 2011), temperature vegetation dryness index, TVDI (Gao et al., 2011), and Normalized Difference Water Index, NDWI (El-Hendawy et al., 2017; Orimoloye et al., 2018c) and Normalized drought Dryness Index, NDDI (Gu et al., 2007; Rhee et al., 2010) were utilized for drought assessment based on land surface temperature, LST and vegetation indices as well as NDDI (Orimoloye et al., 2018b). Hence, scientists have started to concentrate on the reaction of vegetation canopy to drought stress, and likewise on the RS of vegetation as an obscure observation of drought stress.

Cape Town area is witnessing an acute drought as a result of several factors such as climate change and variability, decrease in rainfall amount, inadequate monitoring by environmental stakeholders and significant irrigation supply factors in the area and these may affect agricultural practices and other water-dependent activities. Therefore, crops and agricultural practice might have been severely affected in the period of severe drought. More so, proper monitoring and assessment of drought require more research-based commitment as this can be devastating to people, food security and environmental health if not properly and timely appraised.

However, plants and animals, as well as other features, are influenced by drought events. Various abiotic and biotic elements regain strength when the drought is over while some can never regain again which can lead to their extinction. Thus, droughts can reduce soil quality due to less organic activity, heavy wind erosion and extinction of some soil organisms; drought can also cause water bodies to shrink leading to the death of water animals (Blauhut et al., 2015; Wilhite et al., 2014; Orimoloye et al., 2018c). The recent drought occurrence in South African cities including the study area might increase the devastating state of water dearth in the area and its possible direct impact on agriculture and domestic water usage (Nobre et al., 2016; Yan et al., 2016; Orimoloye and Adigun, 2017). More so, the health and condition of freshwater biomes, for example, ponds, rivers and lakes, wetlands are influenced, with the living organisms in them becoming risked and vulnerable to this environmental risk (Orimoloye et al., 2018c). Animals move long ways of looking for water. Consequently, they migrate to new environments, in most times rendering them exposed to the environmental-related risk, while others experience different hazards and this may lead to biodiversity and ecosystem extinction (Orimoloye et al., 2018c).

The social implication of droughts is perhaps the most felt, as it directly involves individuals and communities. For instance, in many developed countries, days without adequate water can be a nightmare. Human wellbeing has an immediate connection with water availability of any settlement. Clean water for drinking and local use, and in addition sanitation, helps individuals to avert and manage infections (Dale et al., 2001). Studies have suggested that droughts caused low food production especially in low-income nations where the natural source of water is life-reliant and people have less to eat which might result in ill-health and possibly demise (Epstein, 2000; Dale et al., 2001). This is peculiar to remote communities of developing nations, where communication and accessibility are usually inadequate. Furthermore, people migrate from where there is a water dearth to other places in search of better living conditions as a result of drought and this makes the area to be drought vulnerable, as many of its residents are forced to relocate. Farming and other agricultural practices suffer more when people migrate. Droughts have been identified to have more impact on rural areas of the world put pressure on family lives; people feel insecure and are threatened by forest extinction and wildfires there could also be a loss of human life due to drought occurrence in any given area (Leng et al., 2015). This study focused on the landscape dynamics and drought events in Cape Town area, South Africa. In doing so, this study aimed at assessing the drought severity in the study area using Geographic Information System (GIS) and remotely sensed data with high resolution, to quantify the spatial configuration of drought indices in the study area and examine its potential impacts between year 2014 and 2018.

## 2 Materials & methods

**Fig. 1:**
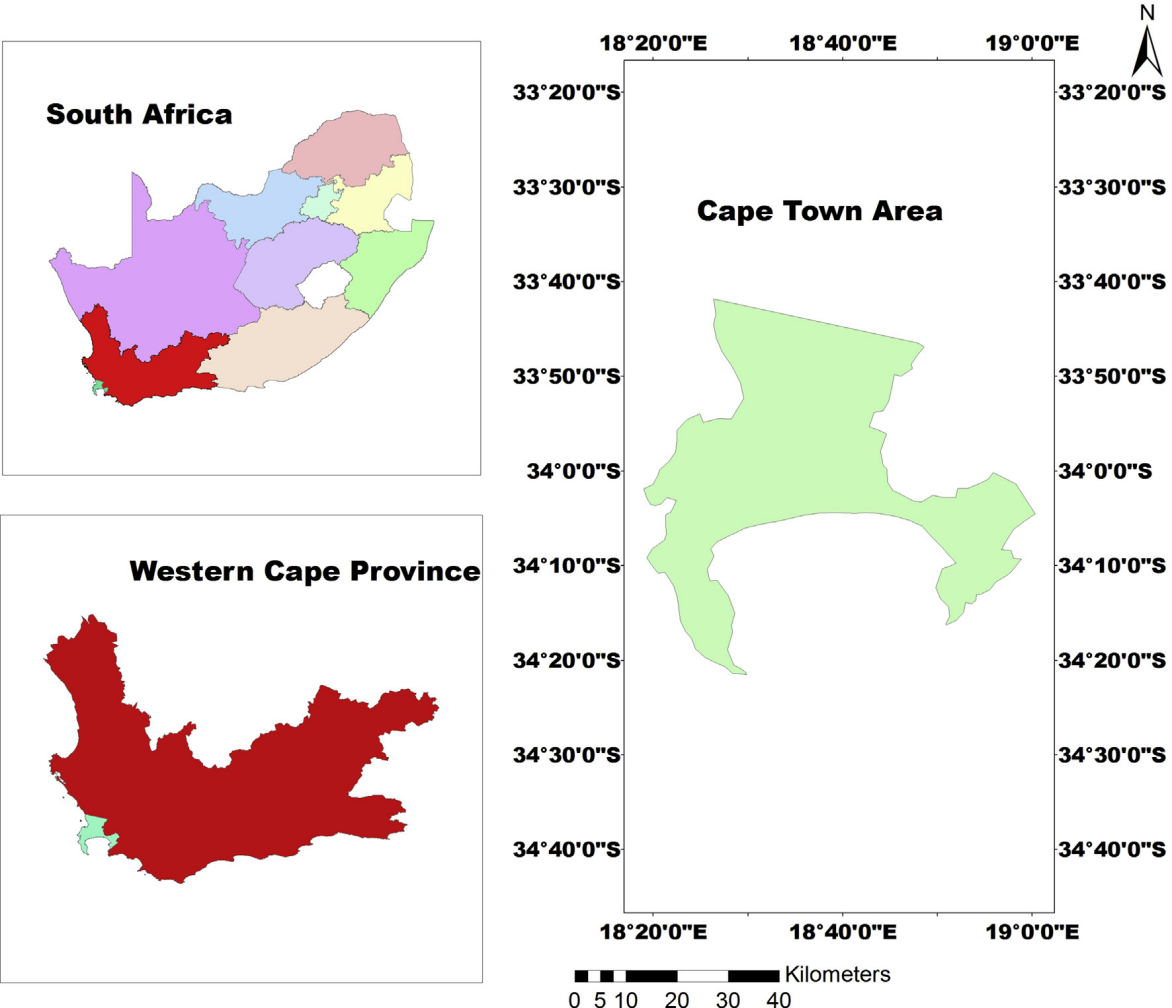
Study area location.

The study area encompasses Cape Town in Western Cape, South Africa (Fig. 1). The city is a port city on the country's southwest coast and has a land cover area of about 400.3 km^2^. It is located at latitude 33.55° S and longitude 18.25° E. The area has a warm Mediterranean climate (Rohli and Vega, 2011) with moderately wet winters, mild and dry, warm summers. The summer, which lasts from late November to March, is warm and dry with an average maximum of 26.0 °C (79 °F) and a minimum of 16.0 °C (61 °F). Winter lasts from the beginning of June to August and may witness large cold fronts entering the inland from the Atlantic Ocean for limited periods with significant precipitation and strong north-westerly winds. Winter months in the city average maximum temperature of 18.0 °C (64 °F) and a minimum temperature of 8.5 °C (47 °F)”. The area experiences a total annual average rainfall of about 515 mm.

The satellite images for the study area during the years 2014–2018 operational land imager (OLI) and thermal infrared sensor (TIRS) Landsat 8 imageries were acquired from the USGS archives (Table 1) and were administered in 1:150.000 shapefile map of Cape Town area. All the imageries were radiometrically rectified (Chander and Markham, 2003; Ogunjobi et al., 2018; Orimoloye et al., 2018b). The years that were chosen for the study based on the onset of severe drought in the study as revealed in previous studies (Leslie and Richman, 2018; Otto et al., 2018; Jordaan et al., 2019).

**Table 1:** Specifications of the satellite images used for the drought assessment.

Data	Year	Date of Acquisition	Path/Row	Thermal lines	Cloud cover_land (%)
Landsat 8 OLI_TIRS	2014	2014-12-05	175/84	7701	6.85
Landsat 8 OLI_TIRS	2015	2015-01-06	175/84	7701	0.09
Landsat 8 OLI_TIRS	2016	2016-12-26	175/84	7701	4.47
Landsat 8 OLI_TIRS	2017	2017-11-27	175/84	7701	0.07
Landsat 8 OLI_TIRS	2018	2018-01-14	175/84	7701	1.69

### 2.1 Image processing

**Fig. 2:**
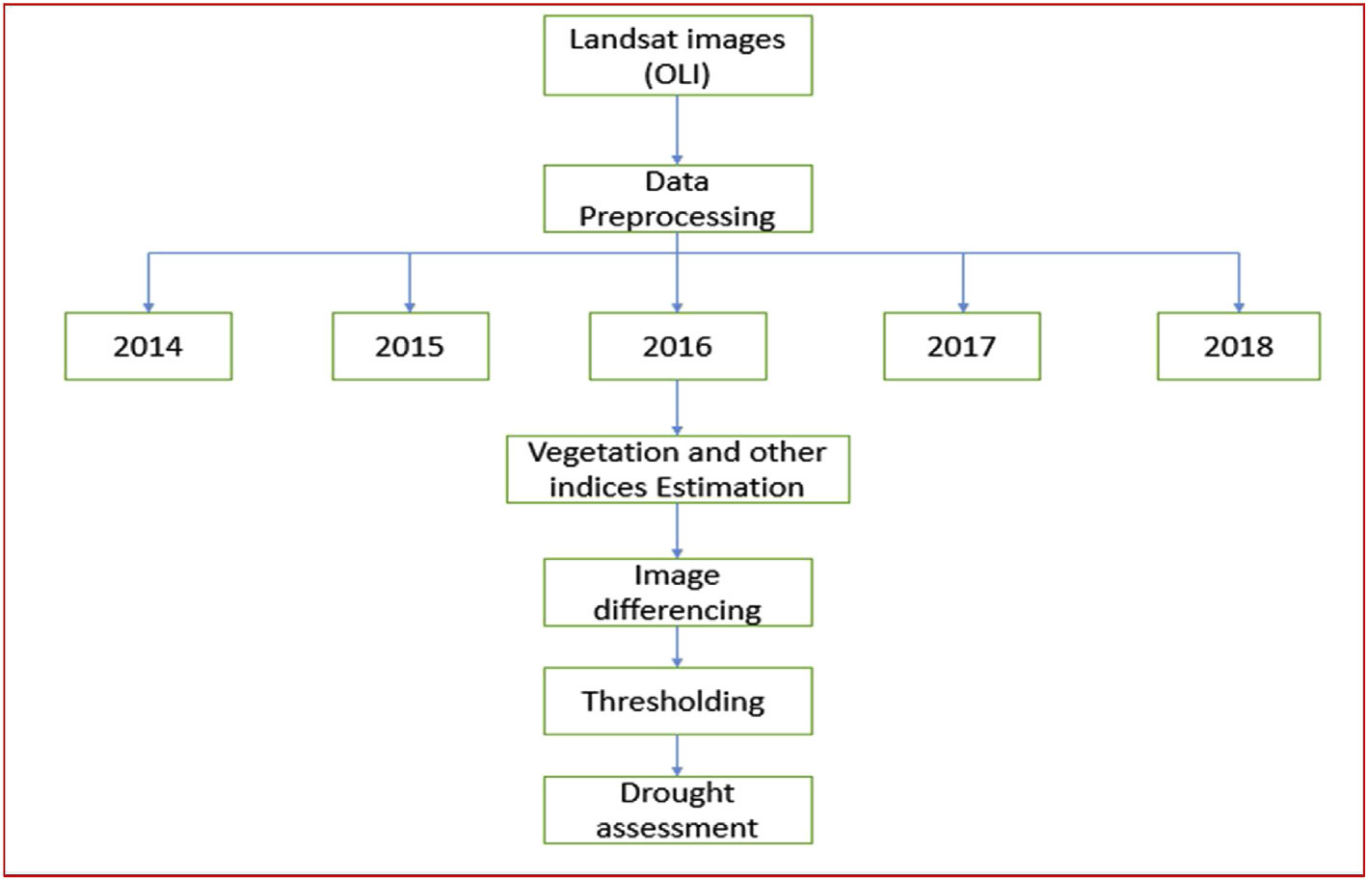
Flow chart of the processes used in this study.

All raw remotely sensed images of the study area were obtained from the USGS database. The satellite imageries collected were in five segments, data in 2014, 2015, 2016, 2017 and 2018. All the data acquired are good quality images of less than 10% cloud cover as presented in Table 1. More so, the information in Fig. 2 and Table 1 show the flow chart and data specifications of the satellite data used for this study, and they are all named after their features such as path and row, date acquired, thermal lines, months and years of acquisition and they were all analyzed with ArcGIS 10.3 GIS tool. “The operational land imager (OLI) image incorporates the shortwave infrared (SWIR) band, thermal infrared (TIR) band, near-infrared (NIR) band and visible bands. TIRS bands are thermal infrared bands with a higher resolution compared with TIR bands (2014–2018 images)”.

**Fig. 3:**
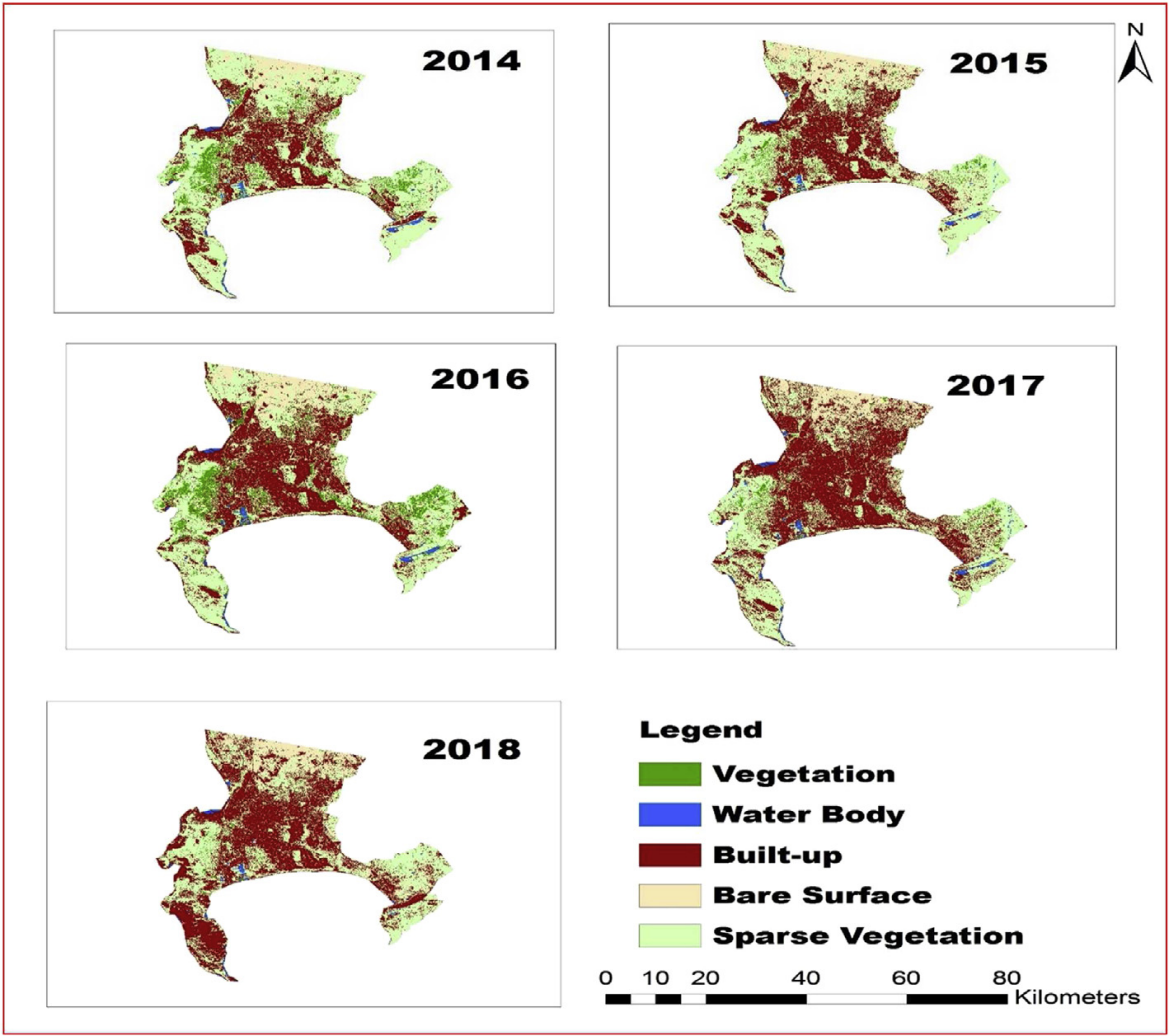
Land use features between 2014 and 2018.

Land cover classes are typically mapped from digital remotely sensed data through the process of supervised digital image classification (Tewkesbury et al., 2015; Yang et al., 2015; Shalaby and Tateishi, 2007). The goal of the image classification process is to automatically categorize all pixels in an image into land cover classes (Shalaby and Tateishi, 2007). The maximum likelihood classifier quantitatively evaluates both the variance and covariance of the category spectral response patterns when classifying an unknown pixel so that it is considered to be one of the most accurate classifiers since it is based on statistical parameters. This study used supervised classification and using ground checkpoints with digital topographic maps of the study area. The area was classified into five main classes: vegetation, water body, built-up, bare surface and sparse vegetation. Descriptions of these land cover classes are presented in Fig. 3.

## 3 Results and discussion

The analysis of supervised classification of the study area between 2014 and 2018 is shown in Fig. 3; and along with the land area coverage for the various land features retrieved from the imagery and drought indices used in this study were presented in Tables 2 and 3 respectively, the area covered with different land features (vegetation, water body, built-up, bare surface (newly cultivated area, sand fill, open surface and rock) and sparse vegetation cover. In year 2014 the study area covered by sparse vegetation was (7382 (42%)) km^2^ followed by built-up area of about (5301 (30%)) km^2^; the area covered by water body was (619 (3%)) km^2^, while bare surface and vegetation cover (2337 (13%)) and (2095 (12%)) km^2^, respectively in the same period. More so, the results revealed that there was a drastic change in vegetation cover between 2014 and 2015; built-up area and vegetation cover area of about (5991 (34%)) and (1438 (8%)) km^2^, respectively, while water body was (279 (1%)) km^2^ lesser than the previous year with about (340) km^2^. Sparse vegetation has the highest area coverage for the year 2015 with about (7747 (44%)) km^2^ and bare surface with (2279 (13%)) km^2^.

**Table 2:** Indices used for drought appraisal in Cape Town area.

SN	Indices	Equation	References
1	LST	*LST =*$\frac{Tb}{1+(\lambda \;*\;Tb\;\left(\rho \right)In\text{ε}\;}$	Williams, 2009, Orimoloye et al. 2018b
2	SATVI	*SATVI =*$\frac{\rho Nir-\;\rho green}{\rho Nir\;+\;\rho green+L}\;\left(1+L\right)-\frac{\rho B7}{2}$	Qi et al., 1994; Qi et al., 2002; Noroozi et al., 2011
3	NDWI	*NDWI =*$\frac{\rho green-\;\rho Nir}{\rho green\;+\;\rho Nir}$	Zhang et al., 2017a,b; Orimoloye et al., 2018c
3	NDDI	*NDDI =*$\frac{NDVI-NDWI}{NDVI+\;NDWI}$	Domingo et al., 2015
4	WDVI	WDVI = *ρNir – γρred* *Where γ = 1.06*	Clevers, 1991
5	L (soil adjusted factor)	*L = 1* – 2 a * NDVI * WDVI	Allbed et al., 2014
6	NDVI	$\frac{\rho Nir-\;\rho Red}{\rho Nir\;+\;\rho Red}$	Yengoh et al., 2015

**Table 3:** Land use dynamics and its percentage between 1986 and 2016.

LULC/Year	2014 (km^2^)	2015 (km^2^)	2016 (km^2^)	2017 (km^2^)	2018 (km^2^)
vegetation	2095 (12%)	1438 (8%)	736 (4%)	353 (2%)	141 (1%)
Water Body	619 (3%)	279 (1%)	527 (3%)	202 (1%)	167 (1%)
Built-up	5301 (30%)	5991 (34%)	6486 (37%)	7930 (45%)	8191 (46%)
Bare Surface	2337 (13%)	2279 (13%)	2722 (15%)	1328 (7%)	1381 (8%)
Sparse vegetation	7382 (42%)	7747 (44%)	7263 (41%)	7921 (45%)	7854 (44%)

Land features characteristics for 2016 as presented in Fig. 3 and Table 3 with the percentage of the area covered for each feature include vegetation, water body, built-up, bare surface and sparse vegetation for the year 2016. Sparse vegetation covers land area of about (7263 (41%)) km^2^ followed by built-up area with (6486 (37%)) km^2^ for year 2016. While water body and bare surface have area coverage of (527 (3%)) and (2722 (15%)) km^2^ respectively, for the same year, vegetation has area coverage of about (736 (4%)) km^2^. In year 2017, vegetation in the study area decreased drastically compare with the previous years where built-up and vegetation area coverage of (7930 (45%)) and (353 (2%)) km^2^ respectively. Water body covers land area of about (202 (1%)) km^2^ while bare surface and vegetation have land cover of about (1328 (7%)) and (353 (2%)) km^2^ respectively, for the same year. Land features characteristics for 2018 revealed changes in land use dynamics with the percentage of the area covered for each feature for the year. There exists a drastic decreased in vegetation, water body coverage in the study area where the water body and vegetation have area coverage of (167 (1%)) and (141 (1%)) km^2^ respectively. While the built up covers land area of about (8191 (46%)) km^2^ followed by sparse vegetation area with about (7854 (44%)) km^2^ for year 2018. While, for the same year, vegetation has area coverage of about (141 (1%)) km^2^.

### 3.1 Vegetation indices

The summary of the characteristics and crucial observations from the five years of drought assessments is presented in this study. This study used LST and five indices (SATVI, NDWI, WDVI, NDDI and NDVI) as presented in Table 2 to assess the drought occurrence in the study area between the years 2014 and 2018.

**Fig. 4:**
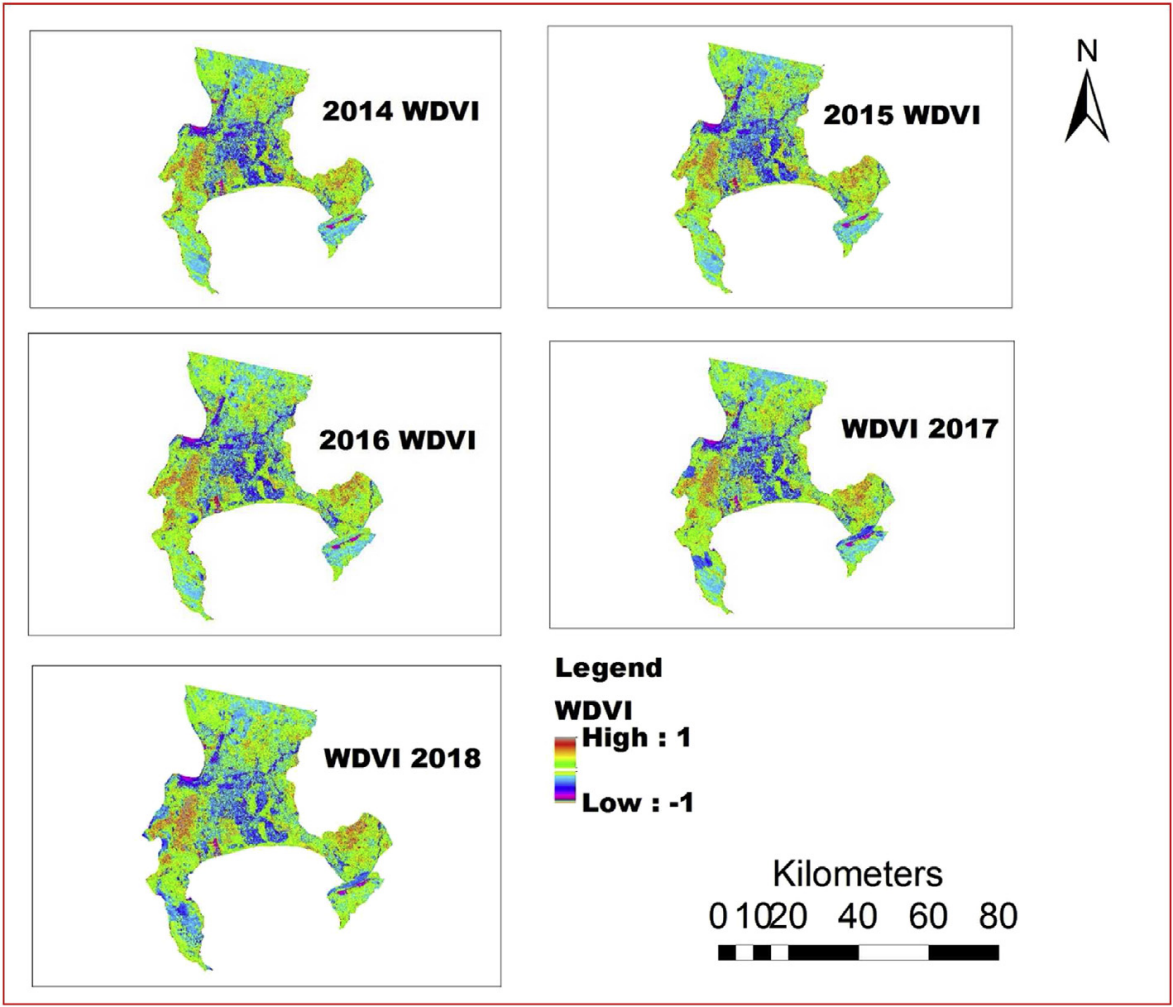
Weighted difference vegetation index for 2014, 2015, 2016, 2017 and 2018.

**Fig. 5:**
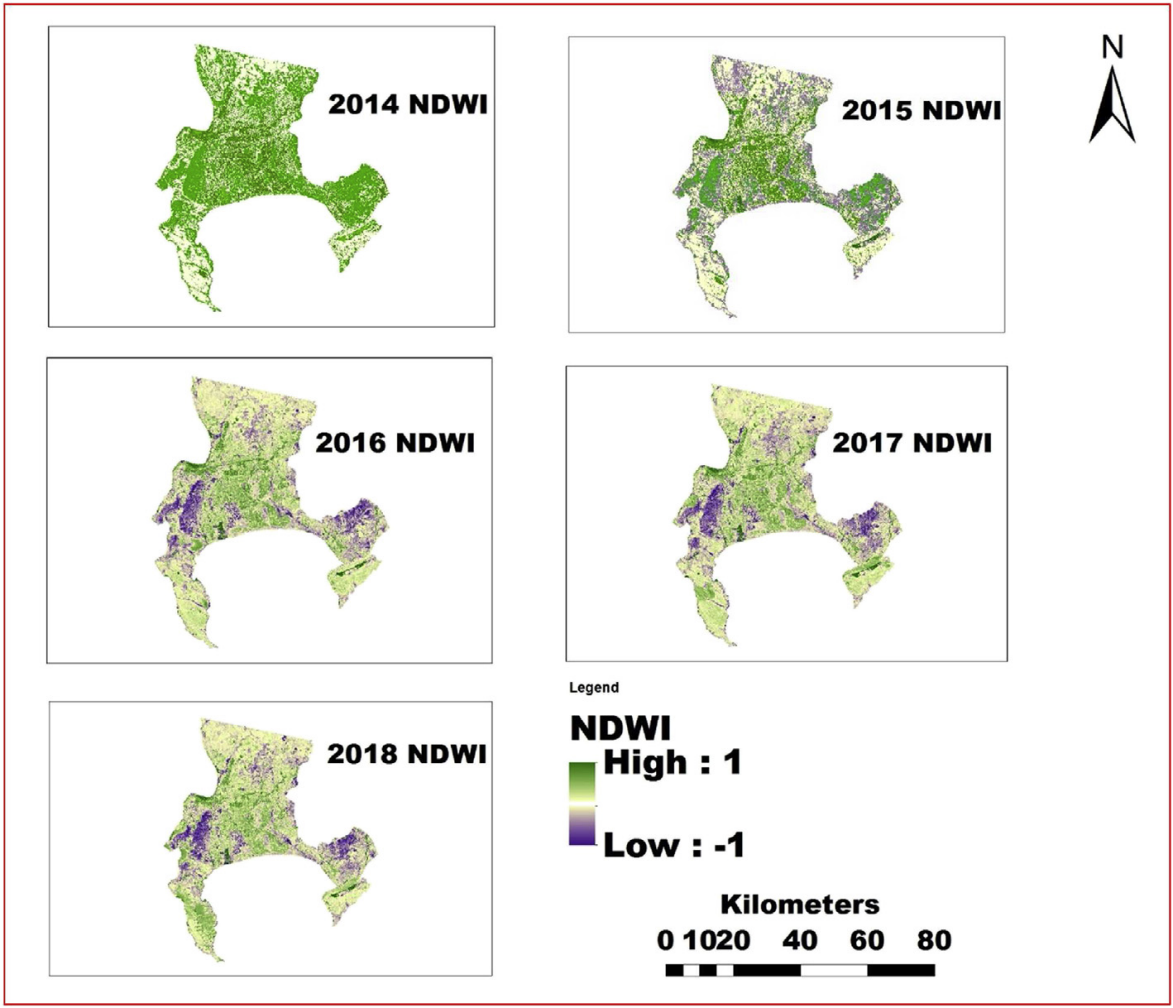
Spatial variation of NDWI of Cape Town area between 2014 and 2018.

**Fig. 6:**
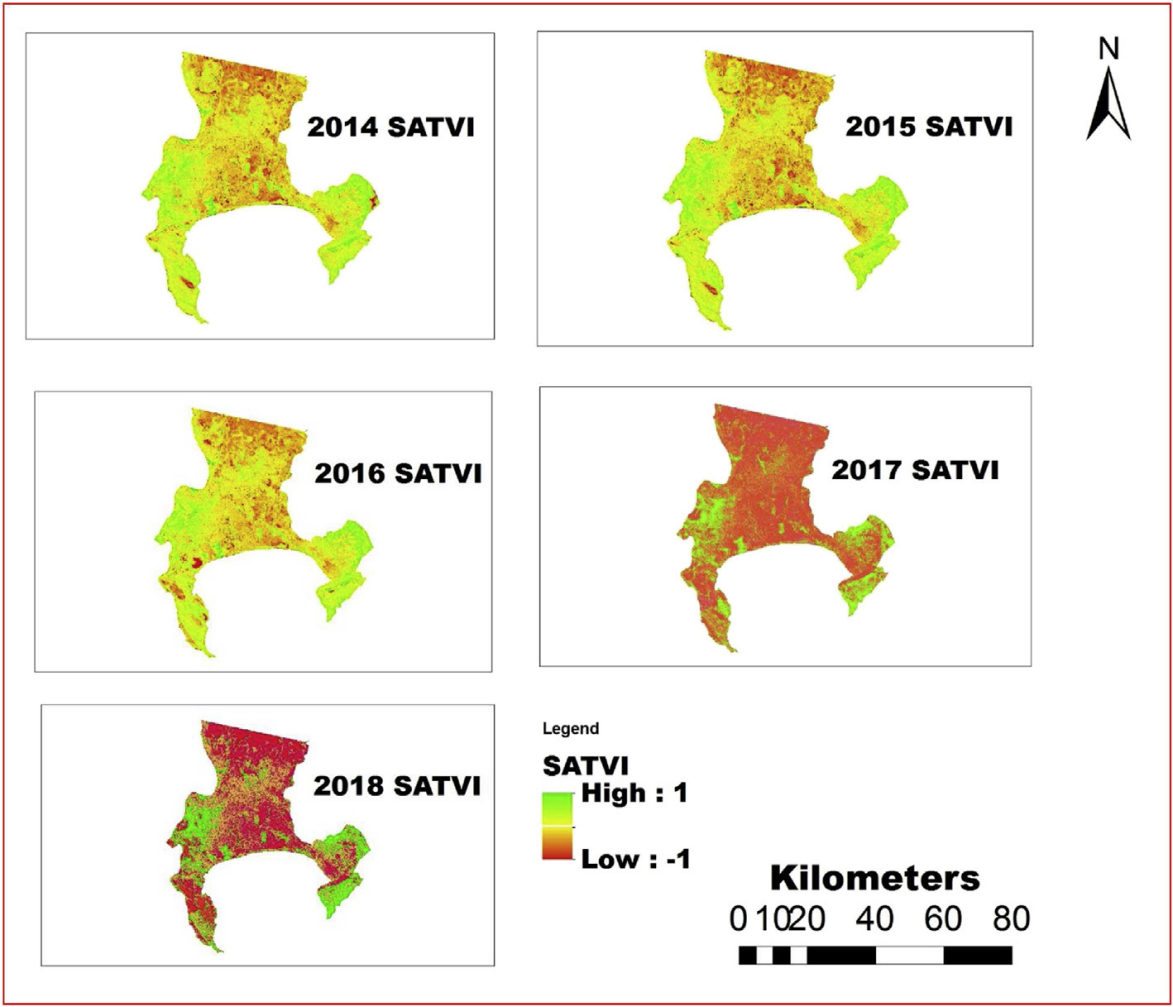
Spatial variation of SATVI of Cape Town area between 2014 and 2018.

**Fig. 7:**
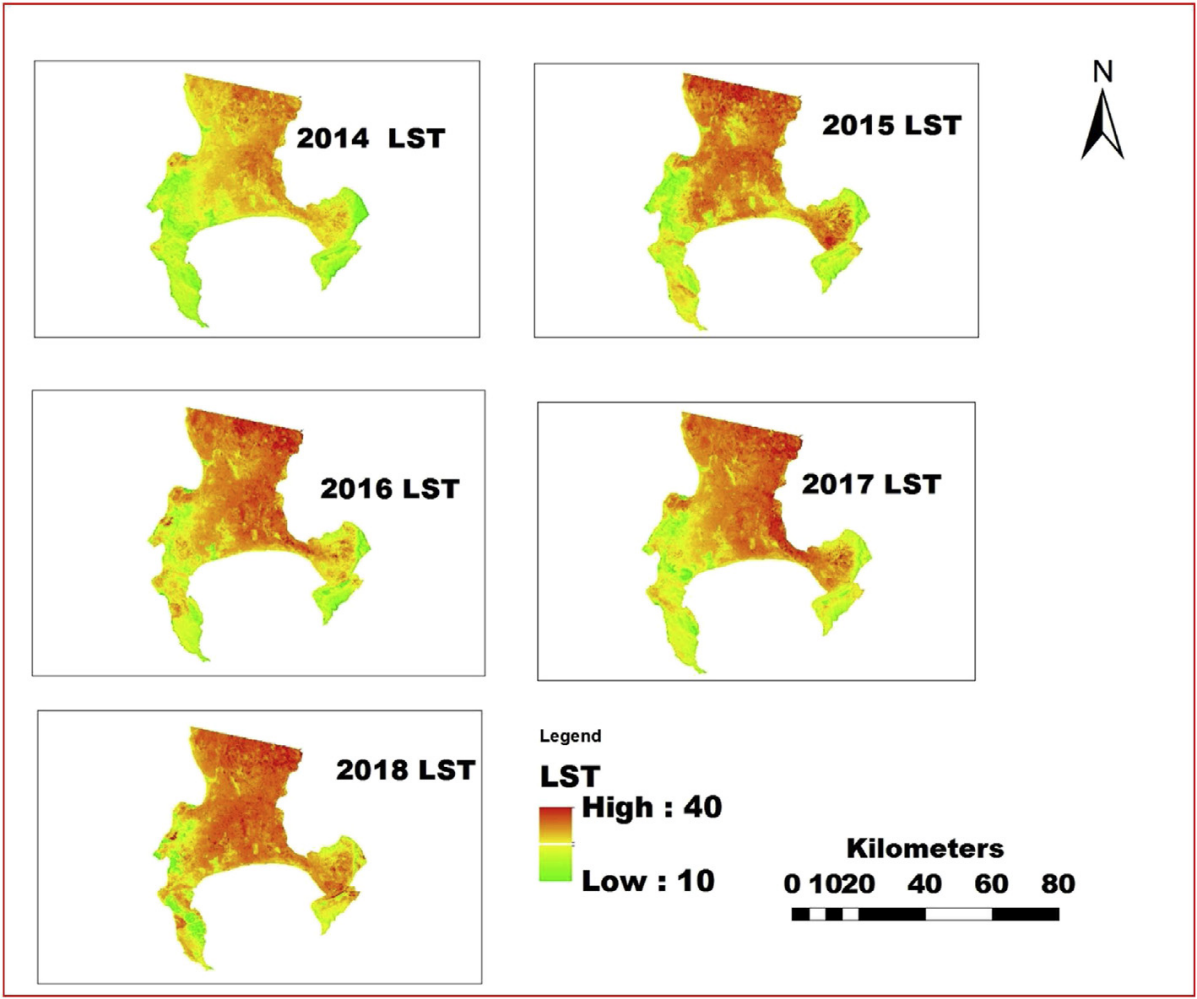
Land surface temperature for 2014, 2015, 2016, 2017 and 2018.

**Fig. 8:**
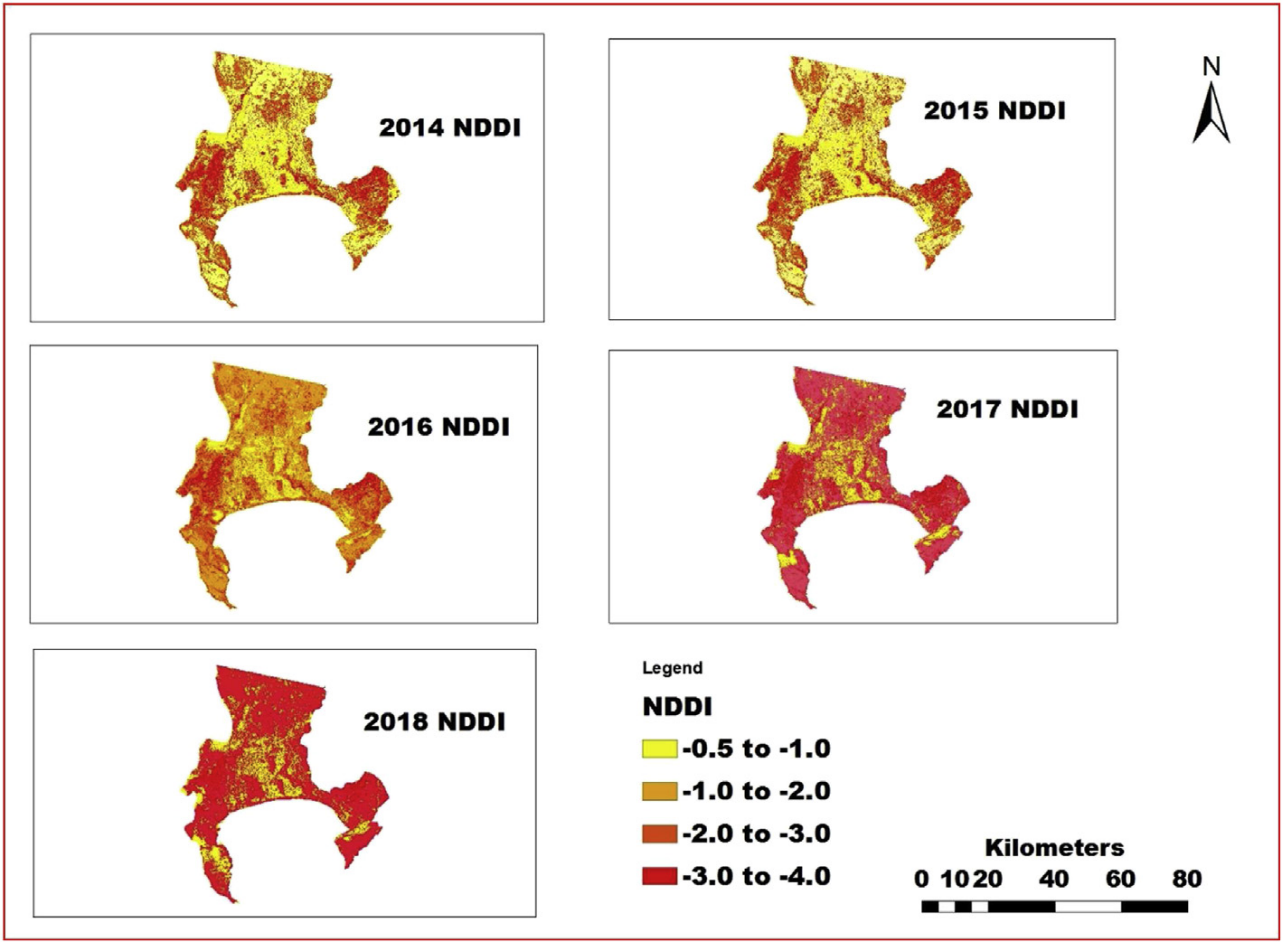
Spatial variation of NDDI in the study area for 2014, 2015, 2016, 2017 and 2018.

**Fig. 9:**
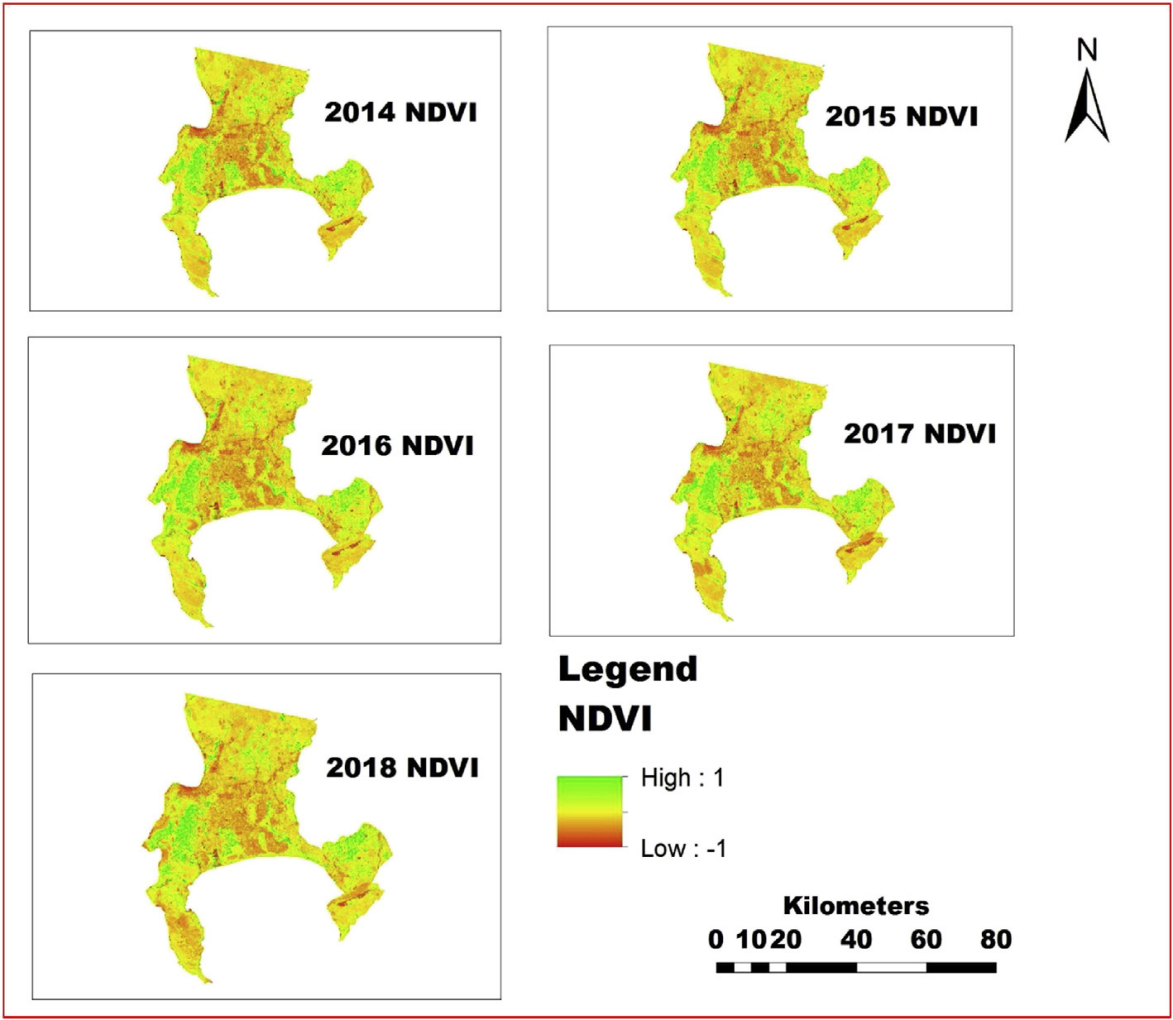
Spatial variation of NDVI for 2014, 2015, 2016, 2017 and 2018.

The information shown in Figs. 4, 5, 6, 7, 8, and 9 reveals the current state of vegetation health in the Cape Town area using satellite images obtained during summer periods (November to January, subject to the availability of the good images for the study area). The variation in the LST and vegetation indices for the summer seasons are evaluated and presented in Figs. 4, 5, 6, 7, 8, and 9. The results from this study show that drought occurs predominantly during the assessed periods as asserted by previous studies that droughts episode in most cases are witnessed in the summer months which was validated by this study (Vicente-Serrano et al., 2014; Diffenbaugh et al., 2015; Wolf et al., 2016). The NDWI and corresponding drought severity designations are presented in Figs. 5 and 8. The results reveal that the areas with low NDWI values are susceptible to drought severity while the areas with high NDWI connotes little or no drought occurrence as asserted by previous investigations (Hanson and Weltzin, 2000; Gu et al., 2007, Park et al., 2016). The NDWI and other indices values decreased considerably more in recent years 2017 and 2018 as revealed in Figs. 4, 5, and 6 which connotes that this development could be a sign of drought occurrences during the period compared to the years 2014–2016. The WDVI-NDWI variation was also moderately intense during the drought situations (December 2016 to January 2018). This increment was more evident in years 2017 and 2018, which corroborates the alarmingly high water dearth and drought occurrence in the area (Berger, 2017; Botai et al., 2017). Evidently, this study has revealed the current state of vegetation health and its implication on drought severity episodes in the study area.

### 3.2 Spatial variation of LST and NDDI

In recent decades, drought has been identified as part of the chronic climate-related hazards happening in many nations in the African continent including South Africa, often with devastating implications on food security (Qi et al., 1994; Sheffield et al., 2014; Klisch and Atzberger, 2016). Using surface reflectance from 2014 to 2018, NDVI, LST and NDDI values were calculated and all remote sensing variable values including LST NDDI and NDVI with 1 km resolution was used as an index of accumulated surface water content were also scaled from 10 to 40 for LST, -4 to -0.5 for NDDI and -1 to 1 (NDVI) for each pixel to discriminate the weather component from the ecosystem component as done by Kogan (1995) for VCI using NDVI. The variations of each drought conditions were examined and the scaling was done so that the scaled value -1 means the driest condition and 1 means the wettest condition for NDVI, greater and equal to 40 °C means the driest condition and less than and equal to 10 °C means the wettest condition for LST and greater and equal to -4 means the driest condition and less than and equal to -.5 means the wettest condition for NDDI during the period. The result further revealed that year 2016, 2017 and 2018 experienced driest conditions as depicted in Figs. 7, 8, and 9, which corroborates previous studies on drought assessment (Jordaan et al., 2019; Leslie and Richman, 2018; Ogunjobi et al., 2018; Otto et al., 2018). The results from the study revealed that NDVI have the opposite direction to NDDI, and LST and the likely reason for the opposite directions is that the NDVI mostly detects soil moisture in the arid region and vegetation moisture in the humid region compared to other indices.

Various vegetation indices have been utilized by different studies to assess water dearth with different shortcomings of these indices (Gu et al., 2007; Rojas et al., 2011; Alshaikh, 2015; Sholihah et al., 2016; Zhang et al., 2017a,b). More so, studies have shown that other vegetation indices are considered to be more efficient than NDVI. For instance, at 40 percent the noise level of the NDVI is 4 times in green cover than that of the WDVI and about 10 times that of the SATVI which is equivalent to a vegetation estimation error of about +/- 23% for the NDVI, while WDVI and SATVI is about +/- 7% and +/- 2.5% estimated error respectively (Qi et al., 1994). Therefore, the WDVI and SATVI are better representative vegetation indicators than the other vegetation indices; more so, other factors such as climatic and geographical zone can also play a crucial role in this development (Qi et al., 1994). All the indices used in the study are indications of drought conditions because they reflect the energy and water exchanges among vegetation, soil, and atmosphere, and considers the characteristics of soil moisture (Rhee et al., 2014).

Land surface temperature (LST) of the study area is presented in Fig. 7; each year has its thermal characteristics and it was depicted from the analysis that the LST was higher in the inner city which is likely to be the built-up region compared to the regions covered with vegetation and other features. The result further shows that there was variation in LST between 2014 and 2018. It also revealed that the built-up area connotes that the urban area has high thermal signal as shown in Fig. 7 compared with the other land features that have a lower land surface temperature probably due to vegetation cover and water body as opined by Peng et al. (2014) and Orimoloye et al. (2018a,b,c). This development might have contributed immensely to the drought occurrence in the area during the period of investigation.

### 3.3 Influence of drought severity on vegetation and other biodiversity

Previous studies have identified the notable impacts of inter-annual precipitation variation on vegetation health such as NDVI and NDWI (Karamihalaki et al., 2016; Khan et al., 2016; Orimoloye et al., 2018c). This study found that in most of the study area, the NDWI values during the peak of highest vegetation covers corroborated with the drought severity assessed using the LST and NDDI (Figs. 7 and 8). The NDDI is most likely suitable for recognizing the implications of water availability and drought situations on vegetation health in semi-arid areas including the study area than only precipitation, as the NDDI encompasses NDVI and NDWI obtained from a reliable and dependable source (satellite data) and considers the active atmospheric evaporative interest area that is, Cape Town area (mild Mediterranean) which has relatively mild winters and very warm summers. More so, the results from the study revealed that the vegetation cover gives more grounded negative relationship with LST, and NDDI for all land features at all levels, while built-up area and open surface give positive relationship of LST and NDDI during the period of investigation. The LST, NDDI and other drought indices pattern fluctuate during the same period but reaches the highest value in the built-up area followed by bare surface. These results connote that the areal measure of vegetation plethora has more direct correspondence with the drought occurrence and the surface thermal characteristics which are the attributes of the land surface that control or influence drought events in any given region. This research also reveals that the major land features that were used in the study area are vegetation.

Recent studies using remote sensing and GIS as well as other techniques have demonstrated that in different locales under current global warming conditions, the warming earth and drought can have a greater influence on the vegetation dynamics than other natural hazards (Hanson and Weltzin, 2000). This is because drought can largely determine the plant and soil-water stress which might adversely affect agricultural practices and in turn have significant effects on food security and human well-being (Turner and Annamalai, 2012; Wheeler and Von Braun, 2013; Nobre et al., 2016; Yan et al., 2016; Orimoloye et al., 2018b, 2018c). Moreover, the NDDI and some other related vegetation indices are evaluated on various factor scales, allowing classification of the best fitting indicator to identify the diverse response times of vegetation alliances to water shortage as a result of drought (Nobre et al., 2016; Yan et al., 2016). The procedures utilized in this study is without a doubt in detecting the drought impacts on vegetation change and other vegetation-based practices such as farming and urban greening system which play a vital role in ameliorating climate change impact (Orimoloye et al., 2018b).

Various studies have established the significance of droughts in engendering land degradation activities (Ibrahim et al., 2015; Yengoh et al., 2015). Nevertheless, the changes in drought severity can be a crucial factor driving land degradation processes and NDWI and NDVI trends in the mild Mediterranean zones. Hence, it is crucial to note that the results obtained in this study advocate that the drought event may not define different changes observed in NDVI (Fig. 9). Consequently, other indices such as SATVI, WDVI, NDWI, and LST can also reveal more drought states as presented in Figs. 4, 5, 6, 7, and 8. The observed spatial patterns of NDDI using Palmer Drought Severity Index (PDSI) as presented in Table 4 revealed that the years 2016–2018 have experienced a severe drought in the study area. PDSI was employed to identify the susceptibility of the study area to drought using NDDI values combined with LST for the chosen period. The illustration of drought classification for PDSI values of Cape Town area is presented in Table 4. The information in Fig. 4 is harmonious with the WDVI values which may be considered to be less efficient in vegetation dryness and drought assessment and has a slight effect on green vegetation cover, rendering it relatively insensate to low vegetation cover as asserted by previous studies (Qi et al., 1994; Wu et al., 2010).

**Table 4:** Discretization criteria for NDDI and Palmer Drought Severity Index categories (Fuchs, 2012).

Drought Class	NDDI Values	Categories
0	0	No drought
D0	-0.5 to -0.99	Incipient dry spell
D1	-1.0 to –1.99	Mild Drought
D2	-2.0 to -2.99	Moderate Drought
D3	-3.0 to -3.99	Severe Drought
D4	-4.0 or -less	Extreme Drought

In support of the results from this study, drought in the Western Cape of South Africa including the study area commenced in 2015 and is leading to a critical water deficit in the area, most strikingly influencing the city of Cape Town. Moreover, in spite of water saving strategies, it was confirmed that dam levels have faced a drastic decline in the past three years (Table 5). After quality rainfall in 2013 as well as 2014, the area ​began to experience dry spell in 2015, followed by three years of dry winters, which were likely linked to El Niño ​events and ​climate variability in the affected area (Araujo et al., 2016; Baudoin et al., 2017). This development also corroborated with the information in Fig. 8 which reveals the drought severity in recent years. The information retrieved from the City of Cape Town's Water Dashboard shows that the level of dam declined from about 71 % to 24 % in years 2014 and 2018 respectively. This drop is highly significant and if it persists, the area can become more vulnerable to extreme dryness which may have negatively influenced the environment and the residents of the area (Leng et al., 2015).

**Table 5:** Water level percentage of total dam capacity by year over the study area (Obtained from the City of Cape Town's Water Dashboard).

Major dams	May 2014	May 2015	May 2016	May 2017	16 February 2018
Wemmershoek Dam	58.8	50.5	48.5	36	48.0
Berg River Dam	90.5	54.0	27.2	32.4	53.4
Steenbras Upper	79.1	57.8	56.9	56.7	83.6
Theewaterskloof Dam	74.5	51.3	31.3	15	11.6
Steenbras Lower	39.6	47.9	37.6	26.5	40.0
Voelvlei Dam	59.5	42.5	21.3	17.2	16.7
Total stored (megalitres)	646 137	450 429	279 954	190 300	220 808
Total ​% Storage	71.9	50.1	31.2	21.2	24.6

The results from this study revealed that the observed patterns and drought situations across the observed period in the study area suggest substantial vulnerability of biodiversity and ecosystems in the area. However, some studies have asserted drought to be a significant element in land degradation processes globally including Africa (Nicholson et al., 1998; Fensholt et al., 2015). This can also affect or contribute to the decrease in agricultural production and other water-dependent activities due to drought severity in recent years in the area. Moreover, it was confirmed that the year 2017 was the driest between 2014 and 2018 as shown in Figs. 5 and 8 and Table 5.

Nevertheless, the effects of drought are not consistent and identified by the availability of water in the soil. Therefore, studies have shown that production would reduce under drought situation due to the independent of water availability and plant physiological activities are significantly compelled by water dearth which may be as a result of severe drought. This could help explain the reason the findings in this study revealed a general negative trend between NDWI and NDDI as well as LST in the study area which was connoted by a negative NDDI pattern with NDWI (for instance, from 2016 to 2018). More so, water deficits could limit agricultural practices and adversely affect the domestic use of water in the area (Leng et al., 2015).

## 4 Conclusion

This study presents a spatially synergistic approach in assessing drought occurrence in Cape Town area of South Africa between 2014 and 2018 using remotely sensed information. The study revealed the importance of RS and GIS in appraising drought severity. However, with the aid of headway in modern RS technologies, comparisons were readily carried out to assess this natural hazard and other possible related environmental disasters. This study used some selected vegetation indices as well as the NDDI and LST to investigate drought occurrence in the study area during the period of study.

This study utilized five land use features include vegetation, built-up, water body, bare surface, and sparse vegetation division estimated from a spectral mixture grouping or classification as an indicator for the purpose of comparison with drought indices. The outcomes revealed that the vegetation fraction gives a more grounded negative connection with LST, and NDDI for all land features at all levels, while built-up area and open surface give positive relationship of LST and NDDI between 2014 and 2018. The LST, NDDI and other drought indices pattern fluctuate with years, but yields the highest value around the built-up area followed by bare surface. These discoveries propose that the areal measure of vegetation plethora has more direct correspondence with the drought, thermal characteristics, and moisture attributes of the surface that control or influence drought events. This research also reveals that the major land features that were used in the study area are vegetation.

The results also reveal that the NDWI and other vegetation indices decreased considerably more in recent years (2017 and 2018) than in the previous years (2014–2016). This development has contributed to the droughts occurrence during the years 2017–2018. Conversely, the spatial trends of LST and NDDI have witnessed increment in recent years, with the NDDI values ranging between moderate drought and severe drought thresholds. Consequently, if this increment persists, such development can have adverse effects on residents in terms of food insecurity, land degradation and environmental health deterioration. The results further suggest that the NDWI and NDDI can be joint to identify vegetation changes as well as drought severity and its potential impact on agriculture and the environment. Findings from the study show that the shortage of water content in the study area in 2017 and 2018 was more severe than in 2014 and 2015. However, utilizing NDWI and NDDI, as well as LST, cannot be used to draw an ultimate conclusion on drought occurrence as other factors can also be incorporated. These factors include soil type, geographic location as well as climate zone. Subsequently, a more thorough evaluation of the vegetation dynamics, drought severity monitoring and meteorological variables incorporated with remotely sensed data are required.

## Declarations

### Author contribution statement

I. Orimoloye, S. Mazinyo, A. Kalumba, W. Nel, O. Ekundayo, A. Afolayan, E. Busayo, O. Ololade: Conceived and designed the experiments; Performed the experiments; Analyzed and interpreted the data; Contributed reagents, materials, analysis tools or data; Wrote the paper.

### Funding statement

This research did not receive any specific grant from funding agencies in the public, commercial, or not-for-profit sectors.

### Competing interest statement

The authors declare no conflict of interest.

### Additional information

No additional information is available for this paper.
